# The sterility, stability and efficacy of repackaged ziv-aflibercept for intravitreal administration

**DOI:** 10.1038/s41598-022-06831-2

**Published:** 2022-02-22

**Authors:** Jakkrit Juhong, Pear Ferreira Pongsachareonnont, Thanapong Somkijrungroj, Apivat Mavichak, Adisai Varadisai, Pajaree Chariyavilaskul, Tanittha Chatsuwan, Thitima Benjachat Suttichet, Kittisak Kulvichit

**Affiliations:** 1 Department of Ophthalmology, Faculty of Medicine, Chulalongkorn University, Bangkok, Thailand; 2 Vitreoretinal Research Unit, Faculty of Medicine, Chulalongkorn University, Bangkok, Thailand; 3 Clinical Pharmacokinetics and Pharmacogenomics Research Unit, Faculty of Medicine, Chulalongkorn University, Bangkok, Thailand; 4 Department of Pharmacology, Faculty of Medicine, Chulalongkorn University, Bangkok, Thailand; 5 Department of Microbiology, Faculty of Medicine, Chulalongkorn University, Bangkok, Thailand; 6 Antimicrobial Resistance and Stewardship Research Unit, Faculty of Medicine, Chulalongkorn University, Bangkok, Thailand; 7 Ophthalmology Department, King Chulalongkorn Memorial Hospital, 1873 Rama 4 road, Pathumwan, Bangkok, 10330 Thailand

**Keywords:** Drug delivery, Immunochemistry

## Abstract

To evaluate the sterility, stability, and efficacy of repackaged ziv-aflibercept in 1-mL plastic tuberculin syringes for intravitreal injection after storage for up to 90 days at controlled (4 °C) and ambient (25.8 °C) temperature. A total of 168 tuberculin-type 1-mL syringes were prepared containing ziv-aflibercept (100 mg/4 mL). Samples were stored at 4 °C and 25.8 °C for 0, 3, 7, 14, 21, 28, 60, and 90 days. At each time point, four samples were evaluated for the stability and binding affinity of anti-VEGF to VEGF (efficacy) using enzyme-linked immunosorbent assays (ELISAs). All samples were analyzed for microbial growth. No microbial growth was obtained from any of the ziv-aflibercept samples during each time point, indicating that the repackaged ziv-aflibercept stored at 4 °C and 25.8 °C remained sterile. ELISA analysis revealed no significant decrease in concentration, and binding affinity was observed, indicating that the stability and efficacy were preserved. However, the concentration of ziv-aflibercept decreased less than the minimum expected concentration of 8 ng/mL after 60 days at 4 °C and after 30 days at 25.8 °C. The repackaged anti-VEGF drug ziv-aflibercept does not lose stability or efficacy and remains uncontaminated if prepared under sterile conditions and stored at 4 °C for up to 60 days or stored at 25.8 °C for up to 30 days.

## Introduction

Vascular endothelial growth factor (VEGF) plays a crucial role in the pathogenesis of neovascular retinal diseases, such as proliferative diabetic retinopathy, neovascular age-related macular degeneration (nAMD) and choroidal neovascularization^1^. Since VEGF promotes new vessel growth (neovascularization) and disrupts the normal process of vascular cell wall formation, if it is not regulated, it may result in tortuous and fragile new blood vessels, vascular leakage, retinal hemorrhages, tractional retinal detachment, and finally subretinal fibrosis^2,3^. Therefore, anti-VEGF drugs have become an effective treatment for retinal disorders related to angiogenesis, as they inhibit the pathogenesis of disease^4,5^.

Aflibercept (Eylea®; Regeneron Pharmaceutical, Inc., Tarrytown, USA and Bayer Healthcare, Berlin, Germany) was approved by the US Food and Drug Administration (US FDA) for treating nAMD in 2011^6^. This drug is a recombinant fusion protein acting as a decoy receptor that binds to all isoforms of human VEGF-A and VEGF-B as well as placental growth factors (PIGFs)^7^. In comparison to ranibizumab, aflibercept has 100-fold higher binding affinity to VEGF-A, needs less regular dosing with equivalent costs^6^, and is noninferior in terms of efficacy and safety in the treatment of nAMD^8^. For diabetic macular edema treatment, the visual outcome after 1 year of aflibercept was superior to those of bevacizumab or ranibizumab^9^ and resulted in more polyp regression than ranibizumab in polypoidal choroidal vasculopathy patients^10^.

Ziv-aflibercept (Zaltrap®, Sanofi-Aventis, Bridgewater, NJ, and Regeneron Pharmaceuticals) has the same molecular structure and mechanism of action as aflibercept^8^ but a lower effective concentration (ziv-aflibercept, 25 mg/mL; aflibercept, 40 mg/mL) and higher osmolarity (ziv-aflibercept, 1000 mOsm/kg; aflibercept, 300 mOsm/kg)^11^. The US FDA first approved ziv-aflibercept on August 4, 2012 for colorectal cancer refractory to oxaliplatin treatment^12^ and has not been approved for intravitreal administration. The drug has been used off-label in several countries since several studies demonstrate that it is safe and effective compared to other anti-VEGF drugs^11,13,14^ and is highly cost effective^15,16^. The comparison between aflibercept and ziv-aflibercept is shown in Table 1.

**Table 1:** Comparison of the two anti-VEGFs: aflibercept vs. Ziv-aflibercept^11^.

Drug (company)	Aflibercept (Regeneron; Bayer)	Ziv-aflibercept (Regeneron; sanofi)
Molecular weight	115 kDa	115 kDa
Osmolarity	300 mOsm/kg	1000 mOsm/kg
Inhibits	VEGF-A; VEGF-B; PlGF	VEGF-A; VEGF-B; PlGF
Vial dose and volume	4 mg in 0.1 mL	100 mg in 4 mL
Vial cost	1085 USD	548 USD
Cost per injection	1085 USD	11 USD
Injection volume	0.05 mL	0.05 mL

Costs according to the Thailand National Drug Information^28^.

Due to the high cost of aflibercept, repackaged ziv-aflibercept would be an attractive choice for facilitating effective treatment of macular disease, especially in low- and middle-income countries. The 4 mL ziv-aflibercept vial could be repackaged into several prefilled plastic syringes (approximately 50–60 doses) and would cost 100 times less than aflibercept. Because ziv-aflibercept is repackaged from a single vial into 50–60 repackaged syringes at once, the remaining ziv-aflibercept may not be completely used and stored for a long time, especially in a small hospital. In some regions, especially low- to middle-income countries, the drug may be repackaged in a large medical center and delivered to a small hospital in a distant region, requiring the repackaged syringes to be stored temporally at ambient temperatures. Although a recent report demonstrated that ziv-aflibercept maintains its sterility, stability, and ability to bind VEGF when stored under refrigerated conditions for 28 days^17^, studies on repackaged ziv-aflibercept under both refrigerated and ambient temperature conditions have not been reported. The purpose of this study was to evaluate the sterility, stability, and efficacy of repackaged ziv-aflibercept under two different temperature conditions (controlled at 4 °C and ambient at 25.8 °C) for up to 90 days. The results of this study provide practical knowledge of repackaged ziv-aflibercept for long-term storage under both temperature conditions.

## Materials and methods

### Materials

Four ziv-aflibercept (100 mg/4 mL) vials were purchased from Sanofi-Aventis (Thailand) Co. Ltd. (Bangkok, Thailand). Tuberculin-type 1-mL syringes were purchased from Terumo Co. Ltd. (Japan). The Eagle Biosciences Aflibercept ELISA Assay Kits and VEGF Human ELISA Kits (Fisher Scientific) were purchased from Thermo Fisher Scientific, Waltham, MA, USA for the stability and efficacy evaluation, respectively.

### Preparation and repackaging of ziv-aflibercept into 1-mL plastic tuberculin syringes

Each of four ziv-aflibercept (100 mg/4 mL) vials was opened under a laminar flow cabinet using an aseptic technique at the Sterile Product Unit, Department of Pharmacy, King Chulalongkorn Memorial Hospital, Thai Red Cross Society between August 2019 and January 2020. Then, to prepare a total of 168 syringes, 50-µL aliquots of ziv-aflibercept were repackaged into 1-mL sterile plastic tuberculin syringes (Terumo, Japan) and capped with a sterile syringe combi-stopper (B. Braun, German). The capped syringes were then sealed in a sterile plastic package and placed in a holding area until they were transported. All of the ziv-aflibercept preparations were drawn up by the same pharmacist.

The samples were randomly allocated into two storage groups (A, refrigerated at 4 °C, and B, ambient temperature). Stability and sterility tests were performed after 0, 3, 7, 14, 21, 28, 60, and 90 days of storage, and the efficacy test was performed after 0, 14, 28, 60, and 90 days of storage (Table 2). Group A samples were kept at 4 °C in the refrigerator. At each time point, four samples were transported to the lab for testing. During transportation, the samples were wrapped in a brown plastic bag (to shield them from light) and placed in a cooler box with an ice cooling gel pack (strictly regulated temperature at 4 °C with digital temperature sensor monitoring).

**Table 2:** Mean concentrations of ziv-aflibercept (ng/mL) at each time point (mean ± SD, N = 64).

Day	Group A	Group B
0	9.66 ± 1.60	8.44 ± 1.20
3	10.63 ± 0.95	10.53 ± 0.71
7	9.91 ± 3.39	10.16 ± 2.01
14	11.92 ± 1.84	7.31 ± 1.33
21	8.02 ± 3.10	7.32 ± 3.06
28	10.34 ± 2.49	9.19 ± 1.69
60	7.91 ± 2.24	8.92 ± 0.66
90	6.39 ± 2.47	4.96 ± 1.09

Anti-VEGF quantification (ng) of ziv-aflibercept was based on a standard curve. The expected concentration was 10 ± 2 ng/mL, according to the initial concentration and subsequent dilutions. Statistical analysis was performed comparing the samples at each time point to the baseline at day 0 and between groups. All comparisons within groups, p value- > 0.05, and between groups, p value- > 0.05.

Unlike group A, the samples in group B were kept at ambient temperature (23.1–28.3 °C, average 25.8 °C). At each time point, four samples were transported to the laboratory for the same tests as group A but without the cooling gel pack. A data logger (RC-51 Data Logger, Elitech, CA, USA) was used to record the temperature in both groups every 15 min. The time from the drug preparation unit to the laboratory was approximately 20 min.

### Sterility test

At each time point, four samples of ziv-aflibercept from both groups (4 °C and 25.8 °C) were transported to the Department of Microbiology at King Chulalongkorn Memorial Hospital for aerobic bacterial culture. Fifty microliters of the stored material was inoculated onto blood agar plates and incubated at 35 ± 2 °C. The plates were examined for bacterial growth daily for a period of 5 days. A positive culture was defined as the presence of bacterial growth on culture plates.

### Stability test

At each time point, four samples from each group were collected and transported to the lab to be analyzed by the technician using a sandwich ELISA technique from Aflibercept ELISA Assay Kit (IG-AA115, Eagle Biosciences Inc., Amherst, NH, USA) to determine the concentrations of unbound ziv-aflibercept in the repackaged syringes. Until processing, all evaluated samples were diluted to 10 ng/mL in assay dilution buffer, and the assay was then performed according to the manufacturer's instructions. Recombinant human (rh)VEGF-A was used as the immobilized molecule on the surface of a 96-well plate. Horseradish peroxidase-conjugated mouse anti-human IgG was used as the detection antibody, which binds to the Fc portion of aflibercept in the sample. The lower limit of quantitation (LLOQ) of this assay was 5 ng/mL. The standard curve of aflibercept was constructed directly proportional to the assay dynamic range (6–200 ng/mL). The optical density (OD) was measured with a photometer at 450 nm. If a solution retained 80–120% (8–12 ng/mL) of its initial concentration, it was considered stable^18^.

### Efficacy test

At each time point, four samples from each group were collected and evaluated for efficacy by testing VEGF binding activity. The evaluated sample was coincubated with rhVEGF165 (PHC9393, Thermo Fisher Scientific, Waltham, MA, USA). Briefly, the ziv-aflibercept sample and rhVEGF were diluted in phosphate-buffered saline (PBS) to 10 mg/mL and 100 ng/mL, respectively. Then, both substances were mixed and coincubated at room temperature for 30 min. The residue-free rhVEGF-165 in each coincubated sample was quantified by ELISA using the human VEGF-ELISA Kit (KHG0111, Thermo Fisher Scientific, Waltham, MA, USA) following the manufacturer’s manual. rhVEGF was used alone as a positive control. Mouse anti-human VEGF was used as the capture antibody, and biotinylated goat anti-human VEGF was used as the detection antibody. The LLOQ of this assay was 5 pg/mL. The OD was measured with a photometer at 450 nm. All ELISAs were performed by the same technician for both the stability and efficacy evaluation.

The percentage of rhVEGF captured in the mixing substance was measured, and the concentration of free rhVEGF not bound to the complex was used to determine the efficacy of ziv-aflibercept. The percentage of rhVEGF-165 consumed in the mixture of ziv-aflibercept and rhVEGF-165 was then calculated from the equation (1):

1 $$100 \, \times \left( {Initial \, \;rhVEGF \, \;165\; \, concentration - Final\; \, rhVEGF\; \, 165\; \, concentration} \right) \div Initial\; \, VEGF\; \, 165\; \, concentration$$

### Statistical analysis

The results are presented as the mean ± standard deviation (SD). Data were analyzed using a linear mixed model with Jamovi v.1.2.3 software (retrieved from https://www.jamovi.org, Sydney, Australia) run on a MAC OS Catalina 10.15.5 (Apple Inc.) computer for the stability and efficacy tests. The normality of the data was tested using the Shapiro–Wilk test. The tested sample factor was the time of storage, which was set to 0 (baseline), 3, 7, 14, 21, 28, 60, and 90 days, and the between-sample factor was the storage temperature (4 °C and 25.8 °C). Statistical significance was defined as a p value < 0.05.

### Presentation

This study has been presented at the 44th Royal College of Ophthalmologists of Thailand Meeting at the Centara Grand Convention Center, Bangkok, Thailand during 24–27 November 2020.

## Results

### Sterility test

The bacterial culture in blood agar showed no growth at all time periods under both temperature conditions, so aliquoting 50 µL of solution into separate 1-mL syringes maintained sterility.

### Stability test

The mean concentrations of ziv-aflibercept (ng/mL) after storage at either temperature condition for each time point are shown in Table 2 (see Supplementary Table S1 for the entire drug stability level at each time point). In both groups, there were no statistically significant differences in the effective concentration of repackaged ziv-aflibercept between each time point and the baseline (p > 0.05) and no statistically significant differences between groups A and B at any time point (p > 0.05), according to the linear mixed model analysis.

### Efficacy test

The efficacy of repackaged ziv-aflibercept was defined as the percentage of rhVEGF-165 consumed in the mixture of ziv-aflibercept and rhVEGF-165 at each time point, as shown in Tables 3 and 4 (see Supplementary Table S2 for the entire drug efficacy level in each time point). The final concentration of rhVEGF-165 was less than 0.5 ng/mL, and the percentage of rhVEGF consumed by ziv-aflibercept was greater than 99% at all time periods in both groups.

**Table 3:** Mean concentrations of rhVEGF-165 and efficacy of ziv-aflibercept in group A.

Sample group A(4 °C)	Initial concentration of rhVEGF-165 (ng/mL)	Initial concentration of ziv-aflibercept (mg/mL)	Mean concentration of final rhVEGF 165 ± SD (ng/mL)	Percentage of rhVEGF consumed by ziv-aflibercept (%)
	A	B	C	D = A − C/100
D 0	100	10	0.33 ± 0.01	99.67 ± 0.01
D 14	100	10	0.31 ± 0.03	99.69 ± 0.03
D 28	100	10	0.34 ± 0.03	99.66 ± 0.03
D 60	100	10	0.31 ± 0.05	99.69 ± 0.05
D 90	100	10	0.34 ± 0.05	99.66 ± 0.05

**Table 4:** Mean concentration of rhVEGF-165 and efficacy of ziv-aflibercept in group B.

Sample Group B(25.8 °C)	Initial concentration of rhVEGF-165 (ng/mL)	Initial concentration of ziv-aflibercept (mg/mL)	Mean concentration of final rhVEGF 165 ± SD (ng/mL)	Percentage of rhVEGF consumed by ziv-aflibercept (%)
	A	B	C	D = A − C/100
D 0	100	10	0.37 ± 0.04	99.63 ± 0.04
D 14	100	10	0.35 ± 0.04	99.65 ± 0.04
D 28	100	10	0.33 ± 0.02	99.67 ± 0.02
D 60	100	10	0.37 ± 0.06	99.63 ± 0.06
D 90	100	10	0.37 ± 0.01	99.63 ± 0.01

**Figure 1:**
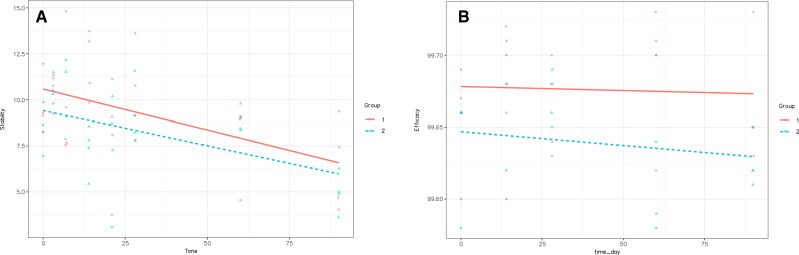
Scatter plot of the ziv-aflibercept (**A**) concentration and (**B**) efficacy in groups A and B over time.

The linear mixed model analysis showed no statistically significant differences in efficacy between ziv-aflibercept stored in sterile plastic tuberculin syringes at 4 °C for up to 90 days compared to the baseline (p > 0.05) or between groups A and B (p > 0.05). Figure 1A,B display scatter plots of the data from Tables 3 and 4 depicting the trends in ziv-aflibercept concentration and efficacy over storage time for groups A and B, respectively.

## Discussion

Neovascular ocular disease is chronic in nature, and most patients require long-term therapy to suppress disease activity, which necessitates 9 to 11 treatment sessions during the first year of treatment (on average, 17 times over 5 years)^19^. As a result, the total cost of treatment is expensive. This reality has a detrimental effect on both patients and healthcare systems, especially in low- and middle-income countries such as Thailand. Repackaged anti-VEGF medications appear to be an attractive option since the dose is precisely administered, and the original anti-VEGF vial could be divided into multiple doses, decreasing waste and cost. However, there is a risk of infection and an increased incidence of endophthalmitis if the vial is punctured multiple times^20^. The repackaged drug should be prepared by a qualified pharmacy at one time and strictly adhere to sterile techniques to reduce the drug contamination risk, prevent endophthalmitis and maintain the efficacy of treatment.

Several studies have reported the sterility, stability, and efficacy of different repackaged or compounding anti-VEGF drugs^17,21–23^. Khalili et al.^24^ reported that the stability of repackaged bevacizumab stored at 5 ± 3 °C is still stable over 6 months. Signorello et al.^25^ confirmed that the sterility and stability of repackaged bevacizumab were maintained if stored at 4 °C and away from UV radiation. Chen et al.^21^ also confirmed that the repackaged bevacizumab stored at 4 °C remained stable for up to 6 months and was sterile. Cao et al.^26^ reported that repackaged ranibizumab and aflibercept in plastic syringes stored for up to 4 weeks does not appear to have a detrimental effect on the in vitro functional activity of these drugs. Sivertsen et al.^27^ revealed that compounded aflibercept can be stored for up to 4 weeks without compromising its quality, stability, or functional properties, including VEGF and neonatal Fc receptor binding. Farah et al.^17^ showed that repackaged aflibercept and ziv-aflibercept do not lose stability or binding affinity and do not become contaminated if prepared under sterile conditions and stored at 4 °C or − 8 °C for 14 or 28 days. As a result of these findings, anti-VEGF drug repackaging is becoming increasingly common in healthcare centers worldwide.

In this study, stability was evaluated by analyzing the effective concentration of ziv-aflibercept using ELISA. We found no significant loss in the concentration of anti-VEGF in either refrigerated (4 °C) or ambient temperature (23.1–28.3 °C) conditions when compared to the baseline for up to 90 days. The mean concentration of repackaged ziv-aflibercept at days 3, 7, 14, and 28 (Table 2) was greater (rather than lower) than that at day 0. The causes of these variations might be pipetting and dilution method errors, as well as the use of different reagents from various batches at each assay throughout the ELISA procedure. However, the variation was between 80 and 120% of the expected concentration (8–12 ng/mL), which was acceptable. Although there was no statistically significant decrease in the anti-VEGF concentration, the concentration continued to drop after 60 days at 4 °C and 30 days at ambient temperature, finally decreasing below the minimum expected concentration (8 ng/mL), as shown in Fig. 1A,B. This observation might be due to the degradation of ziv-aflibercept over time. Therefore, to maintain stability, we recommend using repackaged ziv-aflibercept within 30 days and 60 days if the drugs are stored at room temperature and in the refrigerator, respectively.

The efficacy was also evaluated using ELISA to detect the binding capacity of the anti-VEGF drugs over time after repackaging of the medications. Ziv-aflibercept bound more than 99% of VEGF at all time points, demonstrating that the efficacy was maintained for up to 90 days. The sterility is also maintained.

Our findings support what has been previously reported in the literature, namely, that aseptically repackaged ziv-aflibercept can be stored at 4 °C or 25.8 °C for up to 90 days without losing its sterility, stability, or efficacy. In contrast to most studies evaluating repackaged anti-VEGF medication, the current study also performed a test at ambient temperatures (23.1–28.3 °C, average 25.8 °C). In some region especially low- and middle-income countries, not every hospital can prepare repackaged anti-VEGF drugs because they require a standard laminar flow cabinet, a sterile environmental laboratory and a qualified pharmacist. In some circumstances, the repackaged drug may be prepared in a large medical center and then delivered to a small hospital located in a distant region, requiring the repackaged syringes to be stored temporally at ambient temperature. Because ziv-aflibercept was repackaged from a single vial to prepare 50–60 repackaged syringes at once, the remaining medication may not be used and stored for a long time. Therefore, we evaluated sterility, stability, and efficacy at ambient temperature for up to 90 days to ensure a safe outcome.

One of the strengths of this study include a longer observation time than that of previous studies. In addition, to our knowledge, there are no other studies that evaluated the stability of ziv-aflibercept at ambient temperatures. The limitations of our study include it being a single-center study, the low sample number, and the in- vitro nature of the study. The use of a single blood agar plate for sterility testing is also a disadvantage but was implemented due to the high cost of ziv-aflibercept. However, bacteria can grow on blood agar, which is an enriched medium. Although our result showed that there was no statistically significant loss of stability, efficacy, or sterility of repackaged ziv-aflibercept for up to 90 days, the concentration of VEGF decreased by almost 50% until the storage period of 90 days and the non-significance difference might be due to the low sample size number. Further investigations of greater sample size would be necessary to confirm these finding.

## Conclusion

This study found that there was no significant loss of effective concentration or binding affinity of repackaged ziv-aflibercept for up to 90 days by ELISA analysis. However, the concentration decreased over time and was below the minimum expected concentration when stored at 4 °C after 60 days or 25.8 °C after 30 days. To maintain sterility, stability and efficacy, we suggested using repackaged ziv-aflibercept within 30 days or 60 days if the syringes are stored at room temperature or in a refrigerator, respectively.

## Supplementary Information

Supplementary Information.

## Data Availability

All data generated or analysed during this study are included in this published article (and its Supplementary Information files).
